# Long-Term Intake of* Uncaria rhynchophylla* Reduces S100B and RAGE Protein Levels in Kainic Acid-Induced Epileptic Seizures Rats

**DOI:** 10.1155/2017/9732854

**Published:** 2017-02-23

**Authors:** Nou-Ying Tang, Yi-Wen Lin, Tin-Yun Ho, Chin-Yi Cheng, Chao-Hsiang Chen, Ching-Liang Hsieh

**Affiliations:** ^1^School of Chinese Medicine, College of Chinese Medicine, China Medical University, Taichung 40402, Taiwan; ^2^School of Post-Baccalaureate Chinese Medicine, College of Chinese Medicine, China Medical University, Taichung 40402, Taiwan; ^3^Ko Da Pharmaceutical Co. Ltd, Taoyuan 324, Taiwan; ^4^Graduate Institute of Pharmacognosy, Taipei Medical University, Taipei 110, Taiwan; ^5^Department of Chinese Medicine, China Medical University Hospital, Taichung 40447, Taiwan; ^6^Graduate Institute of Integrated Medicine, College of Chinese Medicine, China Medical University, Taichung 40402, Taiwan; ^7^Graduate Institute of Acupuncture Science, College of Chinese Medicine, China Medical University, Taichung 40402, Taiwan; ^8^Research Center for Chinese Medicine and Acupuncture, China Medical University, Taichung 40402, Taiwan

## Abstract

Epileptic seizures are crucial clinical manifestations of recurrent neuronal discharges in the brain. An imbalance between the excitatory and inhibitory neuronal discharges causes brain damage and cell loss. Herbal medicines offer alternative treatment options for epilepsy because of their low cost and few side effects. We established a rat epilepsy model by injecting kainic acid (KA, 12 mg/kg, i.p.) and subsequently investigated the effect of* Uncaria rhynchophylla* (UR) and its underlying mechanisms. Electroencephalogram and epileptic behaviors revealed that the KA injection induced epileptic seizures. Following KA injection, S100B levels increased in the hippocampus. This phenomenon was attenuated by the oral administration of UR and valproic acid (VA, 250 mg/kg). Both drugs significantly reversed receptor potentiation for advanced glycation end product proteins. Rats with KA-induced epilepsy exhibited no increase in the expression of metabotropic glutamate receptor 3, monocyte chemoattractant protein 1, and chemokine receptor type 2, which play a role in inflammation. Our results provide novel and detailed mechanisms, explaining the role of UR in KA-induced epileptic seizures in hippocampal CA1 neurons.

## 1. Introduction

Seizures are often diagnosed as a neurological disease, with a prevalence of approximately 1%. They are always accompanied by inflammation and oxidative stress [1–4]. Kainic acid (KA) is a potent neuroexcitatory amino acid to activate kainate receptor in the brain and is typically used for developing an animal epilepsy model [5–7]. The KA-induced epileptic seizure model is widely used to exhibit symptoms similar to those of seizures in the human temporal lobe [8–10]. An imbalance of excitatory glutamate and inhibitory *γ*-aminobutyric acid (GABA) receptors, induces uncontrolled electric discharges in the central nervous system (CNS), thus leading to epilepsy [11, 12]. Studies on antiepileptic drugs, namely, topiramate [13] and gabapentin [14], have reported a reduction in the excitatory neurotransmitter level and an increase in GABA biosynthesis, respectively. More than 30% of clinical patients with epilepsy cannot be treated adequately with traditional antiepileptic drugs because of the resultant side effects [15]. Our previous study indicated that auricular electroacupuncture can attenuate epilepsy by altering the TRPA1, pPKC*α*, pPKC*ε*, and pERk1/2 signaling pathways in KA-induced epilepsy in rats [16].

The S100 proteins are calcium-regulated low-molecular weight proteins and were first reported in the brain [17].* The S100 protein family consists of 24 members only expressed in vertebrates and with cell-specific expression patterns [17]*. The S100B subtype is the most abundant in the CNS, melanocytes, chondrocytes, and adipocytes [17–19]. S100B has been well documented for its role in enhancing neurite outgrowth [20, 21]. The potentiation of S100B expression is typically involved in chronic epilepsy [22]. A previous study reported that oral* Uncaria rhynchophylla* (UR) reduced KA-induced epileptic seizures and neuronal death and also lowered S100B proteins levels in rats [23].

The receptor for advanced glycation end products (RAGE), a transmembrane receptor, is upregulated in temporal lobe epilepsy and contributes to experimental seizures [24]. RAGE activation initiates downstream cellular responses such as inflammation and cell proliferation, migration, and differentiation [24].* RAGE has been found to be involved in inflammatory processes [25–27] and is increased in neurons and glia after brain injury [28–31]*. The inflammatory activation of damage-associated molecules, including S100 proteins, can further activate RAGE, resulting in acute and chronic diseases [32]. RAGE regulates NF-*κ*B and ERK signaling resulting from tissue inflammation [33].

Metabotropic glutamate receptors (mGluRs) are subdivided into three groups of G-protein coupled receptors: Group I mGluRs are mainly located at postsynaptic neuron whereas groups II (mGluR3) and III mGluRs are* predominantly expressed* at presynaptic sites to regulate release of neurotransmitters [34]. Recent evidences suggest that mGluR3 plays a crucial role in regulating glutamatergic transmission at both presynaptic and postsynaptic sites in the hippocampus [35].

Monocyte chemotactic protein-1 (MCP-1) is a mediator of inflammation released by activated monocytes and fibroblasts. In addition, MCP-1 is activated through lipopolysaccharide or cytokine stimulation [36, 37]. Gong et al. (1997) indicated that MCP-1 is significantly involved in inflammation, particularly in arthritic inflammation. Haringman et al. (2006) reported that MCP-1 is crucial in leukocyte migration and inflammatory disorders [38, 39]. MCP-1 has frequently been reported to play a role in inflammatory diseases. Wu et al. (2012) reported that MCP-1 plays a crucial role in the migration of neural progenitor cells during neuroinflammation [40]. Furthermore, MCP-1 can promote mesenchymal stem cell migration in vitro and can be blocked by an antagonist [41].

The chemokine receptor type 2 (CCR-2) is a G-protein coupled receptor that is majorly involved with and expressed in inflammatory cells, such as monocytes, neutrophils, basophils, and T-lymphocytes, and in CNS neurons [42–44]. CCR-2 can be activated by inflammatory factors, including chemokines and interleukins [40, 45]. Sandblad et al. (2015) reported on 20 chemokine receptors on 3 monocyte subsets and suggested that CCR-2 is highly expressed in classical (CD14^+^ CD16^−^) but not in nonclassical (CD14^+^ CD16^+^) monocytes [46].

UR has been suggested to exert an anticonvulsant effect in KA-induced epileptic seizures in rats [23, 47]. The constituents of UR, namely,* rhynchophylline*,* isorhynchophylline*, and* isocorynoxeine*, exert neuroprotective effects by reducing glutamate-mediated neuronal loss in cerebellar granule cells [48]. Furthermore, UR reduces apoptosis and exerts a neuroprotective effect [47] by inhibiting c-Jun N-terminal kinase phosphorylation and nuclear factor-*κ*B (NF-*κ*B) activity in KA-induced epileptic seizures in rats [49].

To determine the detailed mechanisms of UR in KA-induced epileptic seizures, we investigated whether UR alters S100B, RAGE, mGluR3, MCP-1, and CCR-2 protein levels 6 weeks after KA injection. We demonstrated that oral UR attenuated the overexpression of S100B proteins, in a similar manner to the positive control by VA. Moreover, we indicated that the RAGE receptor,* a possible downstream target of S100B*, is increased in rats after KA injection and further reduced by UR administration, which is indicative of its therapeutic effect. Similar results were not observed with mGluR3, MCP-1, and CCR-2 proteins. In the current study, oral UR was found to reduce availability of* S100B to the RAGE pathway* in epileptic seizures.

## 2. Materials and Methods

### 2.1. Animals

Male Sprague–Dawley (SD) rats (200–300 g) were used in this study. All rats* had* free access to food and water. Animal use was approved by the Institutional Animal Care and Use Committee of China Medical University and followed the Guide for the Use of Laboratory Animals (National Academy Press).

### 2.2. Extraction of* Uncaria rhynchophylla*

**Figure 1:**
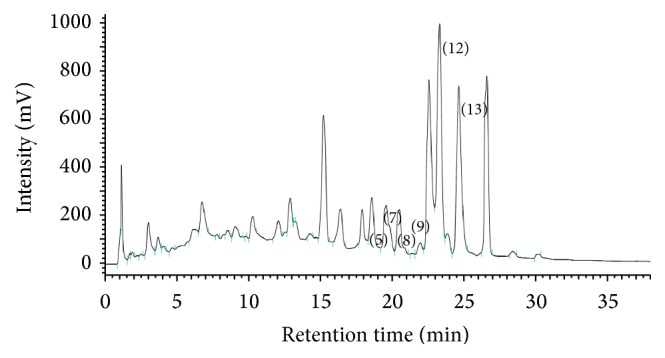
High-performance liquid chromatography (HPLC) fingerprint of* Uncaria rhynchophylla*. The peaks at Rt 17.5 to 25.5 min can be assigned to isocorynoxeine (5), rhynchophylline (7), corynoxeine (8), isorhynchophylline (9), hirsuteine (12), and hirsutine (13).

UR (Rubiaceae,* Uncaria rhynchophylla *[Miq.] Jacks.) was purchased from China and authenticated and extracted by Koda Pharmaceutical Company (Taoyuan, Taiwan), a reputable pharmaceutical manufacturing factory located in Taiwan. Extraction of UR was performed as described in previous studies [49, 50]. In brief, 8 kg of crude UR was extracted with 64 kg of 70% alcohol by boiling for 35 min. These extracts were filtered, freeze-dried, and subsequently stored in a dryer box to obtain a total yield of 566.63 g (7.08%). The freeze-dried extracts were stored at 4°C. The freeze-dried UR extracts were tested for quality with a high-performance liquid chromatography (HPLC) system (interface D-700, Pump L-7100, UV-Vis Detector L-7420; Hitachi Instruments Service Co. Ltd., Ibaraki-ken, Japan) by using* rhynchophylline* (Matsuura Yakugyo Co. Ltd., Japan) as the standard, which was obtained from Koda Pharmaceutical Company. The HPLC fingerprint of UR was analyzed by Koda Pharmaceutical Company as described in our previous study [48] and was shown in Figure 1. Each gram of freeze-dried extract contained 1.81 mg of the pure alkaloid component of UR. The dose response for this compound was reported in our previous study [51]. Hence, this effective dose was used for all of the experiments in the current study.

### 2.3. Establishment of the Epileptic Seizure Model

Twenty-four SD rats (*n* = 6 for all groups) were used in this study. Four days prior to electroencephalogram (EEG) and electromyogram (EMG) recordings, all rats underwent stereotactic surgery following anesthesia with chloral hydrate (400 mg/kg, i.p.). The scalp was incised from the midline, and the skull was exposed. Stainless-steel screw electrodes were implanted on the dura mater over the bilateral sensorimotor cortices to serve as recording electrodes. A reference electrode was placed in the frontal sinus. Bipolar electrical wires were placed on the neck muscles for EMG recordings. Electrodes were connected to an EEG and EMG-monitoring machine (MP100WSW, BIOPAC System, Inc., CA, USA). The epileptic seizures were confirmed with observations of behavioral changes, including wet-dog shakes, paw tremors, and facial myoclonia, under a freely moving and conscious state and epileptiform discharges on EEG recordings. Rats with more than 250 wet-dog shakes and those with the number of facial myoclonia plus paw tremors ≥100 were selected. The rats were randomly divided into 4 experimental groups, as follows: (1) the control group containing rats that received only phosphate buffered saline (PBS) i.p. without KA; (2) the KA group containing rats that received KA (12 mg/kg, i.p.) only; (3) the UR group containing rats that received oral UR (1 g/kg, 5 d/wk) continuously for 6 weeks, starting the day after KA injection; and (4) the VA group containing rats that received oral VA (250 mg/kg/d, 5 d/wk) continuously for 6 weeks, starting the day after KA injection. All rats were sacrificed on day 42 after KA injection, and the brains were removed and divided into left and right brains. Left brain was for immunohistochemistry staining and right brain was for western blot analysis.

### 2.4. Immunohistochemistry Staining

Animals were heavily anesthetized with chloral hydrate (400 mg/kg, i.p.) and perfused with normal saline through the cardiac vascular system, followed by 4% paraformaldehyde (Merck, Frankfurt, Germany) in 0.1 M PBS (pH 7.4). The brains were removed and postfixed in the same fixative overnight at 4°C. After brief washing with PBS, the brains were transferred to a 30% sucrose solution in 0.01 M PBS for cryoprotection, and coronal sections containing the hippocampal area were cut into 20 *μ*m thick slices through cryosectioning. The sections were preincubated for 2 h at 25°C with 10% horse serum and 0.3% Triton X-100 in PBS to avoid nonspecific binding. The sections were incubated overnight at 4°C with the primary antibody CCR2 (1 : 1000, Abcam, UK), mGluR3 (1 : 1000, Abcam, UK), MCP-1 (1 : 1000, Abcam, UK), RAGE (1 : 1000, Abcam, UK), S100B (1 : 1000, Novus Biologicals, USA), Actin (1 : 1000, Millipore, USA), 0.1% horse serum, and 0.1% Triton X-100 in PBS. The sections were subsequently incubated for 2 h at 25°C with biotinylated-conjugated secondary antibody (diluted at 1 : 200; Vector, Burlingame, CA 94010, USA), followed by incubation with avidin-horseradish peroxidase complex (ABC-Elite, Vector). The sections were finally visualized with 3,3′-diaminobenzidine as the chromogen. Sections were washed 3 times with PBS during the incubation steps for 10 min per cycle. The stained hippocampal slices were sealed under the coverslips and then examined for the presence of immune-positive hippocampal neurons using a microscope (Olympus, BX-51, Japan). The immune-positive signals were quantified with NIH ImageJ software (Bethesda, MD, USA).

### 2.5. Western Blot Analysis

Right hippocampi were excised immediately for protein extraction. Total protein was prepared by homogenizing the hippocampi in a lysis buffer containing 20 mmol/L of imidazole-HCl (pH 6.8), 100 mmol/L of KCl, 2 mmol/L of MgCl_2_, 20 mmol/L of EGTA (pH 7.0), 300 mmol/L of sucrose, 1 mmol/L of NaF, 1 mmol/L of sodium vanadate, 1 mmol/L of sodium molybdate, 0.2% Triton X-100, and a proteinase inhibitor cocktail for 1 h at 4°C. From each sample, 30 *μ*g of proteins were extracted and analyzed through a BCA protein assay. They were subjected to 7.5%–10% SDS-Tris glycine gel electrophoresis and transferred to a nitrocellulose membrane. The membrane was blocked with 5% nonfat milk in a TBST buffer (10 mmol/L of Tris, pH 7.5, 100 mmol/L of NaCl, and 0.1% Tween 20), incubated with the primary antibody in TBST with bovine serum albumin for 1 h at room temperature. Peroxidase-conjugated secondary antibody (1 : 500) was used as the secondary antibody. The membrane was developed using the ECL-Plus protein detection kit. Where applicable, the image intensities of specific bands were quantified with NIH ImageJ software (Bethesda, MD, USA).

### 2.6. Statistical Analysis

All data were presented as the mean ± standard deviation. Statistical significance between the PBS, control, UR, and VA groups was analyzed through one-way ANOVA, followed by Tukey's post hoc test. A *P* value < 0.05 was considered statistically significant.

## 3. Results

### 3.1. Induction of Epileptic Seizures after KA Injection Using EEG

**Figure 2:**
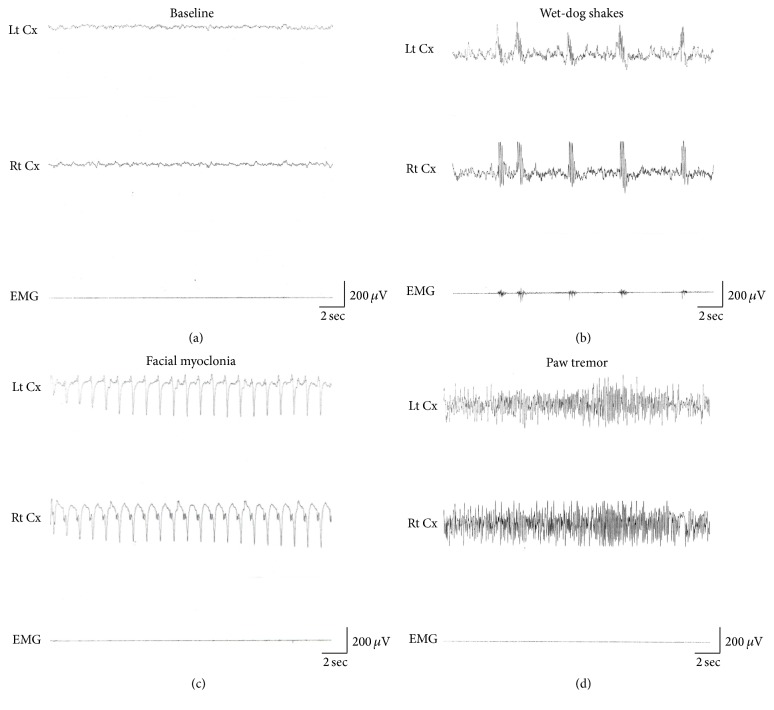
Alteration of electroencephalographic (EEG) signals in KA-injected rats. Baseline EEG activity in the sensorimotor cortex was characterized by 6–8 Hz activity in rats when awake (a). KA-induced temporal lobe seizures, including wet-dog shakes (WDS) with intermittent polyspike-like activity (b), facial myoclonia with continuous sharp waves (c), and paw tremor (PT) with continuous-spike activity (d). Each type of seizures had its own characteristic EEG activity. Lt Cx: EEG recording of the left sensorimotor cortex; Rt Cx: EEG recording of the right sensorimotor cortex; EMG: EMG recording of the neck muscles.

An epileptic seizure is a crucial sign of clinical epilepsy resulting from electric discharges in the brain. In the current study, we injected KA (12 mg/kg, i.p.) to establish an in vivo epileptic rat model. Twenty-four SD rats were injected with KA for inducing epileptic seizures. Three major symptoms of seizures were observed, and their own characteristic electrophysiological activities were recorded. Limbic motor signs such as wet-dog shakes, paw tremor, and facial myoclonia were recorded as the symptoms of epilepsy.  Figure 2(a) shows the baseline conditions. Wet-dog shakes were defined as a polyspike-like EEG activity (Figure 2(b)). Facial myoclonia was defined as a characteristic continuous sharp EEG activity (Figure 2(c)). Paw tremors were characterized by a continuous-spike EEG activity (Figure 2(d)). These parameters were used as an adequate induction of epileptic seizures, and these rats were used for investigating the effect of UR on epilepsy in all of the experiments. The number of wet-dog shakes, facial myoclonia, and paw tremors were significantly different from controls but not between each other. (all *P* < 0.05, Table 1).

### 3.2. Oral UR Reduces S100B Enhancement in KA-Induced Epilepsy

**Figure 3:**
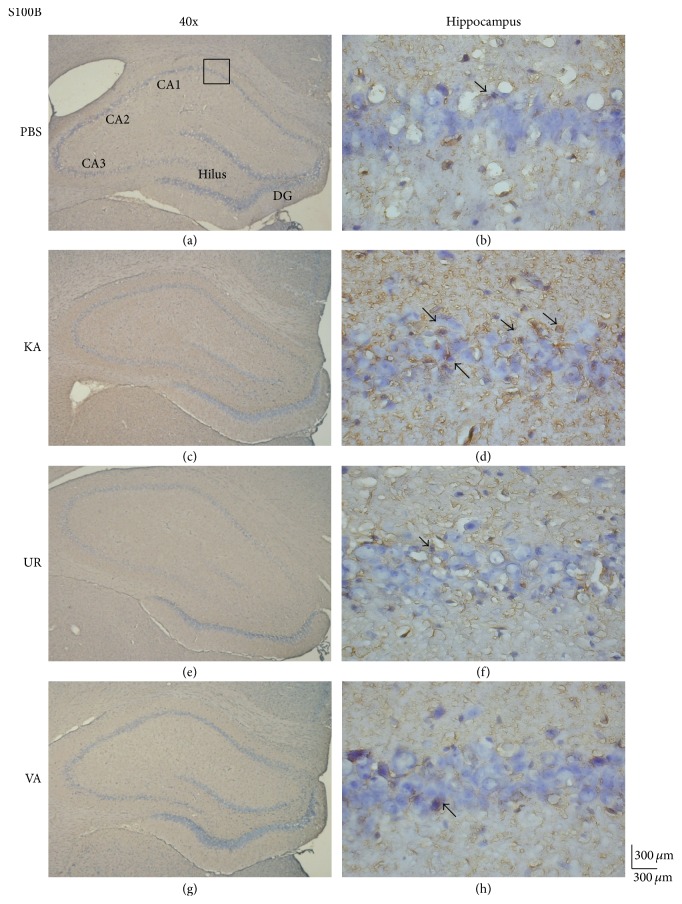
Immunohistochemistry staining of S100B and hematoxylin and eosin (HE) staining in hippocampal sections from the PBS, KA, UR, and VA-pretreated groups. HE (blue) and S100B (brown) immunostaining in the whole hippocampus (a) and CA1 (b) in the PBS group. HE (blue) and S100B (brown) immunostaining in the whole hippocampus (c) and CA1 (d) areas in the KA group. HE (blue) and S100B (brown) immunostaining in the whole hippocampus (e) and CA1 (f) areas in the UR group. HE (blue) and S100B (brown) immunostaining in the whole hippocampus (g) and CA1 (h) areas in the VA group. The left panel was imaged at 40x magnification, and the right panel was imaged at 400x magnification.

We previously reported that oral UR reduces the number of epileptic seizures and neuronal death [23]. In the current study, we attempted to determine whether a 6-week long-term administration of UR reduces S100B expression. S100B proteins were detected in the hippocampus of rats that received PBS injection (Figure 3(a), 97.0 ± 14.39 cells/field, *n* = 6). Specifically, KA injection increased the expression of S100B proteins significantly in the hippocampal CA1 area (Figure 3(b), 260.67 ± 34.89 cells/field, *n* = 6, *P* < 0.05, compared with the PBS group). These elevated levels were further attenuated following UR administration (Figure 3(c), 129.17 ± 19.38 cells/field, *n* = 6, *P* < 0.05, compared with the KA group). Similar observations were reported after the oral administration of VA, a positive control for epilepsy treatment (Figure 3(d), 112.33 ± 20.92 cells/field, *n* = 6, *P* < 0.05, compared with the KA group).

**Figure 4:**
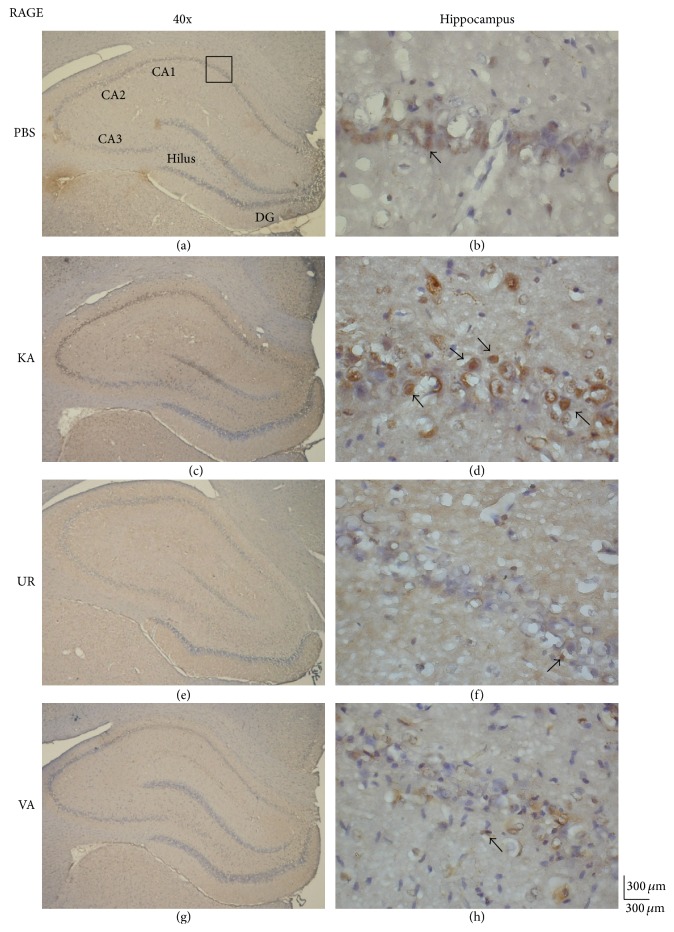
Immunohistochemistry staining of RAGE and hematoxylin and eosin (HE) staining in hippocampal sections from the PBS, KA, UR, and VA-pretreated groups. HE (blue) and RAGE (brown) immunostaining in the whole hippocampus (a) and CA1 (b) in the PBS group. HE (blue) and RAGE (brown) immunostaining in the whole hippocampus (c) and CA1 (d) areas in the KA group. HE (blue) and RAGE (brown) immunostaining in the whole hippocampus (e) and CA1 (f) areas in the UR group. HE (blue) and RAGE (brown) immunostaining in the whole hippocampus (g) and CA1 (h) areas in the VA group. The left panel was imaged at 40x magnification, and the right panel was imaged at 400x magnification.

We next analyzed whether oral UR alleviates the overexpression of RAGE in hippocampal neurons after KA injection. We determined that PBS injection did not induce RAGE potentiation (Figure 4(a), 143.17 ± 38.11 cells/field, *n* = 6). After KA injection, the levels of RAGE proteins in the hippocampus increased substantially (Figure 4(b), 247.67 ± 84.1 cells/field, *n* = 6, *P* < 0.05, compared with the PBS group). These phenomena were attenuated following the administration of UR (Figure 4(c), 100.5 ± 27.59 cells/field, *n* = 6, *P* < 0.05, compared with the KA group) and VA (Figure 4(d), 102.5 ± 32.25 cells/field, *n* = 6, *P* < 0.05, compared with the KA group), suggesting that RAGE plays a critical role in controlling epileptic seizures.

**Figure 5:**
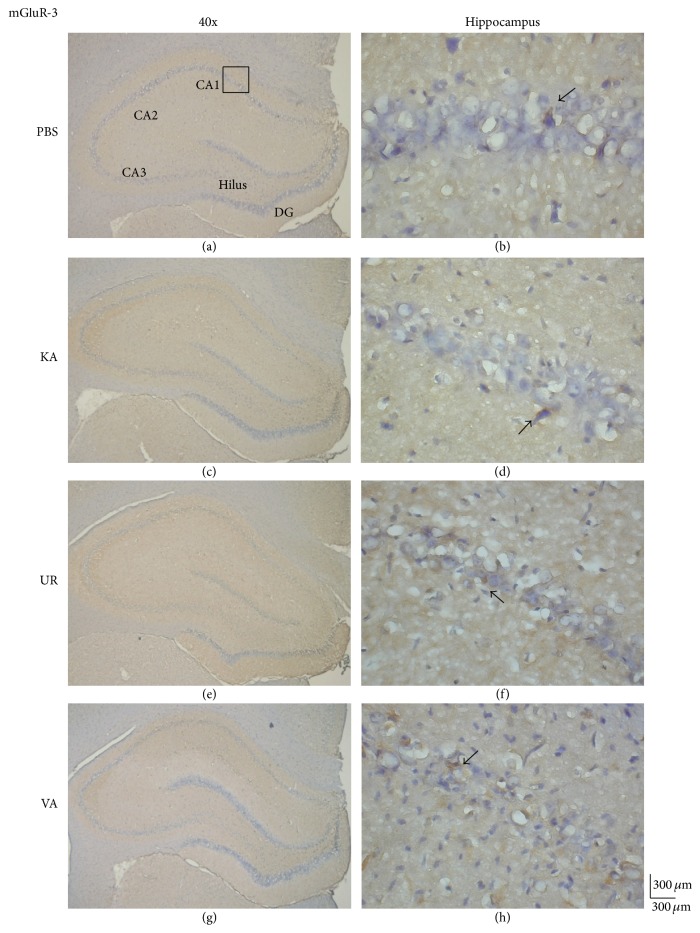
Immunohistochemistry staining of mGluR3 and hematoxylin and eosin (HE) staining in hippocampal sections from the PBS, KA, UR, and VA-pretreated groups. HE (blue) and mGluR3 (brown) immunostaining in the whole hippocampus (a) and CA1 (b) in the PBS group. HE (blue) and mGluR3 (brown) immunostaining in the whole hippocampus (c) and CA1 (d) areas in the KA group. HE (blue) and mGluR3 (brown) immunostaining in the whole hippocampus (e) and CA1 (f) areas in the UR group. HE (blue) and mGluR3 (brown) immunostaining in the whole hippocampus (g) and CA1 (h) areas in the VA group. The left panel was imaged at 40x magnification, and the right panel was imaged at 400x magnification.

We investigated whether mGluR3, an excitatory receptor that is highly associated with S100B activation, was involved in KA-induced epileptic seizures. Our results revealed that the levels of mGluR3 proteins were similar in the hippocampus following the injection of both PBS (Figure 5(a), 91.83 ± 11.79 cells/field, *n* = 6) and KA (Figure 5(b), 97.17 ± 36.64 cells/field, *n* = 6, *P* > 0.05, compared with the PBS group), indicating a negative role in this process. Similar results were obtained for the UR (Figure 5(c), 84.17 ± 10.11 cells/field, *n* = 6, *P* > 0.05, compared with the KA group) and VA groups (Figure 5(d), 87.17 ± 22.53 cells/field, *n* = 6, *P* > 0.05, compared with the KA group). These data suggested that the expression of mGluR3 proteins was not influenced by KA injection alone or KA injection followed by UR or VA treatment.

**Figure 6:**
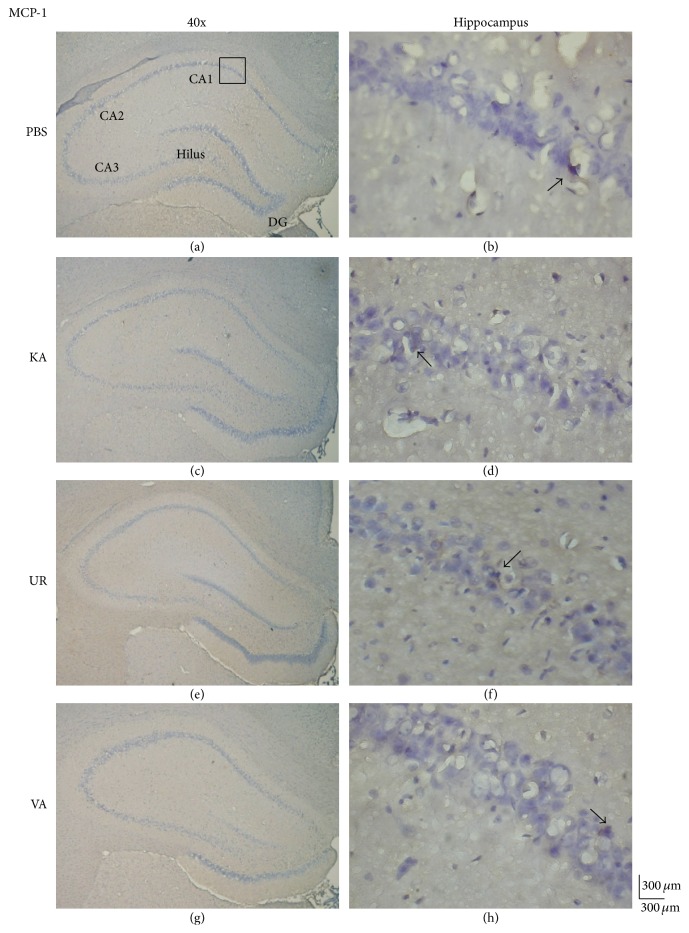
Immunohistochemistry staining of MCP-1 and hematoxylin and eosin (HE) staining in hippocampal sections from the PBS, KA, UR, and VA-pretreated groups. HE (blue) and MCP-1 (brown) immunostaining in whole hippocampus (a) and CA1 (b) in the PBS group. HE (blue) and MCP-1 (brown) immunostaining in whole hippocampus (c) and CA1 (d) areas in the KA group. HE (blue) and MCP-1 (brown) immunostaining in whole hippocampus (e) and CA1 (f) areas in the UR group. HE (blue) and MCP-1 (brown) immunostaining in whole hippocampus (g) and CA1 (h) areas in the VA group. The left panel was imaged at 40x magnification, and the right panel was imaged at 400x magnification.

**Figure 7:**
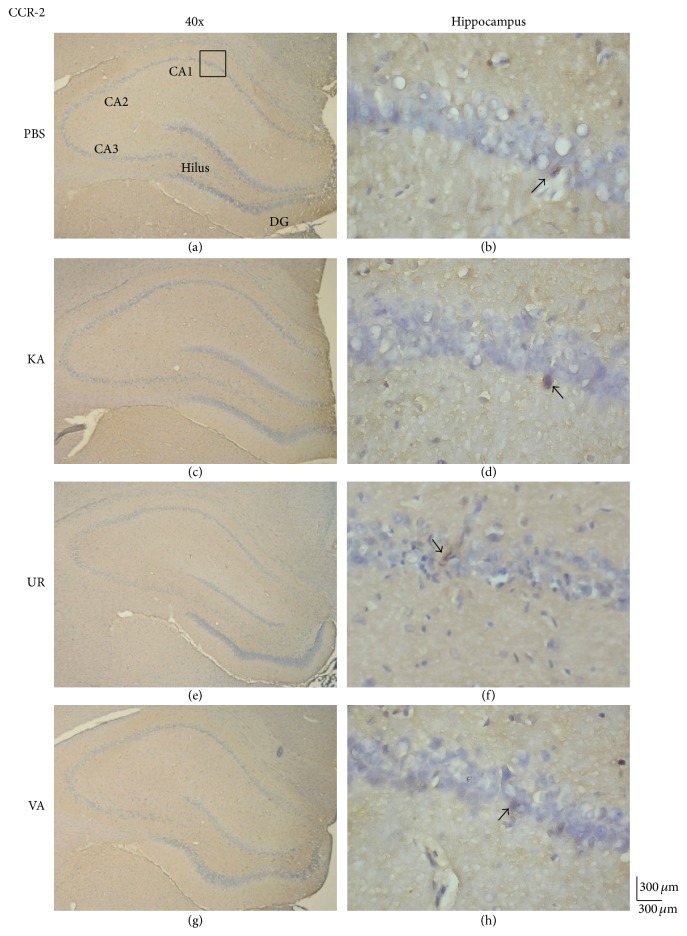
Immunohistochemistry staining of CCR-2 and hematoxylin and eosin (HE) staining in hippocampal slices from PBS, KA, UR, and VA-pretreated groups. HE (blue) and CCR-2 (brown) immunostaining in whole hippocampus (a) and CA1 (b) in PBS group. HE (blue) and CCR-2 (brown) immunostaining in whole hippocampus (c) and CA1 (d) areas in KA group. HE (blue) and CCR-2 (brown) immunostaining in whole hippocampus (e) and CA1 (f) areas in UR group. HE (blue) and CCR-2 (brown) immunostaining in whole hippocampus (g) and CA1 (h) areas in VA group. The left panel was imaged at 40x magnification while the right panel was at 400x magnification.

We investigated whether the level of MCP-1, an inflammation-related molecule, was altered in KA-induced epileptic seizures. The results revealed the presence of MCP-1 proteins in the hippocampus (Figure 6(a), 34.83 ± 17.87 cells/field, *n* = 6) and unaltered levels of MCP-1 proteins 6 weeks following KA injection (Figure 6(b), 42.83 ± 33.63 cells/field, *n* = 6, *P* > 0.05, compared with the PBS group). Furthermore, the 6-week administration of oral UR did not alter MCP-1 expression (Figure 6(c), 47.5 ± 20.95 cells/field, *n* = 6, *P* > 0.05, compared with the KA group). The administration of VA did not exert any effects on the levels of MCP-1 proteins in the hippocampus (Figure 6(d), 41.47 ± 19.47 cells/field, *n* = 6, *P* > 0.05, compared with the KA group). We further investigated whether CCR-2, a chemokine receptor for MCP-1 that is reportedly involved in acute and chronic inflammation, participated in KA-induced epileptic seizures. CCR-2 levels were detected in the hippocampus (Figure 7(a), 56.83 ± 9.6 cells/field, *n* = 6) and were unaltered in the KA group 6 weeks after KA injection (Figure 7(b), 54.5 ± 6.89 cells/field, *n* = 6, *P* > 0.05, compared with the PBS group). Oral UR did not affect CCR-2 expression (Figure 7(c), 54.17 ± 14.41 cells/field, *n* = 6, *P* > 0.05, compared with the KA group). Oral VA also did not affect CCR-2 expression in the rat hippocampus (Figure 7(d), 50.0 ± 2.53 cells/field, *n* = 6, *P* > 0.05, compared with the KA group).

### 3.3. KA Injection Increases Expression of S-100B and RAGE and Is Attenuated by Oral UR and VA

**Figure 8:**
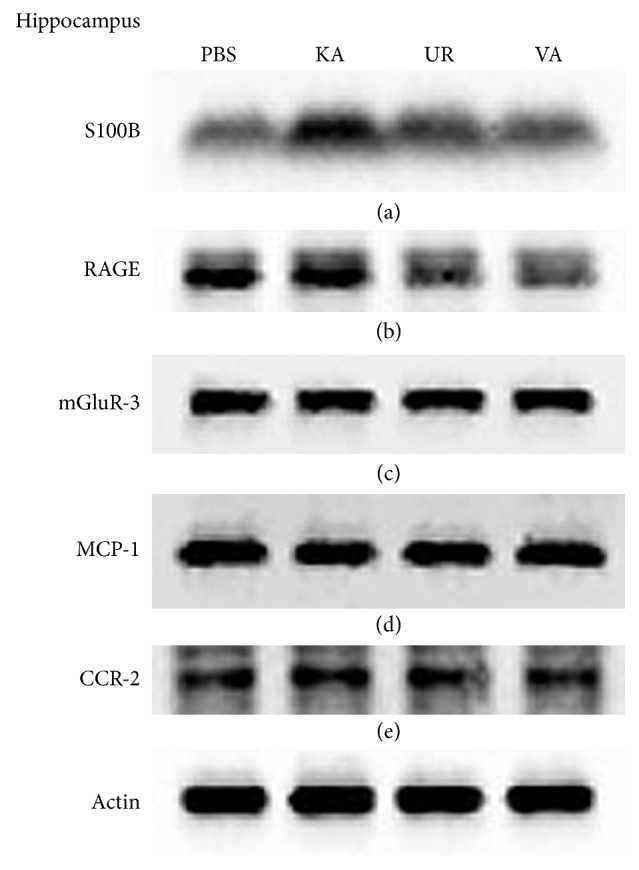
Western blot analysis of S100B, RAGE, mGluR3, MCP-1, and CCR-2 in the hippocampus from the PBS, KA, UR, and VA-pretreated groups. (a) S100B; (b) RAGE; (c) mGluR3; (d) MCP-1; (e) CCR-2. *β*-Actin was set as an internal control.

In our previous study, we demonstrated that oral UR attenuates the overexpression of S100B and RAGE in the rat hippocampus. We further examined this pattern by performing western blotting for quantifying protein levels. Our results revealed that S100B increased in rats following KA injection (Figure 8, 1.44 ± 0.06 versus 1.07 ± 0.10 density, *n* = 3, compared with the PBS group) and can be reversed with the oral administration of UR (Figure 8, 1.25 ± 0.08 density, *n* = 3, *P* < 0.05, compared with the KA group) and VA (Figure 8, 1.10 ± 0.09 density, *n* = 3, *P* < 0.05, compared with the KA group). In a similar manner, RAGE levels increased in the band density in the KA group (Figure 8, 0.81 ± 0.1 versus 0.70 ± 0.06 density, *n* = 3, compared with the PBS group) but returned to baseline after the administration of UR (Figure 8, 0.59 ± 0.05 density, *n* = 3, *P* < 0.05, compared with the KA group) and VA (Figure 8, 0.54 ± 0.08 density, *n* = 3, *P* < 0.05, compared with the KA group). Similar results in the levels of mGluR3, MCP-1, and CCR-2 proteins (Figure 8) were not observed. These data presented strong evidence that oral UR alleviated overexpression in the S100B and RAGE signaling pathway, rather than of mGluR3, MCP-1, and CCR-2 proteins.

## 4. Discussion

In this study, we established a rat epileptic seizure model via i.p. injection of KA and monitored the symptoms by EEG. Three major phenotypes of seizures, namely, wet-dog shakes, paw tremors, and facial myoclonia, were recorded. We used these rats to determine the therapeutic effects and detailed mechanisms of oral UR. We hypothesized that S100B and target receptor RAGE increased after KA injection and were further alleviated by oral administration of UR as well as VA, the positive control. This phenomenon was not observed with the expression of excitatory mGluR3 receptor proteins, suggesting that it did not play a role in KA-initiated epileptic seizures. Furthermore, the administration of UR and VA did not reduce MCP-1 overexpression and the associated downregulation of CCR-2 significantly. This novel finding indicated a crucial effect of UR and underlying mechanisms.

UR is a Chinese medicinal plant with sedative and anticonvulsive effects in epilepsy. An alkaloidal extract of UR contains 5 constituents, namely,* rhynchophylline*,* isorhynchophylline*,* corynoxeine*,* hirsutine*, and* hirsuteine*.* Rhynchophylline* is an oxindole moiety that protects rat neuronal cells from the N-methyl-D-aspartate (NMDA)-induced neurotoxicity [48]. The number of NMDA-mediated neuronal deaths and the expression of apoptosis-relevant genes in the hippocampal sections increased [52]. The administration of the alkaloid fraction of UR in cultured hippocampal neurons alleviates neuronal death and apoptosis-associated genes including* c-jun*,* bax*, and* p53* [53, 54]. We suggested in our previous studies that UR lowers KA-induced lipid peroxide levels [51] and offers neuroprotection against KA-induced epilepsy by regulating GFAP and S100B [23]. Other studies have reported that brain trauma induces temporal lobe damage and causes temporal lobe epilepsy, hippocampal sclerosis, and neocortical damage [55, 56]. We also reported in our previous studies that oral UR prevents death of hippocampal neurons and downregulates the overexpression of glial cells. Moreover, we suggested that S100B protein is potentiated after KA injection, and this phenomenon can be attenuated by oral UR 2 weeks after KA injection. In the current study, we observed that 6-week treatment with oral UR reduced S100B overexpression, suggesting its long-term therapeutic effect. In addition, we demonstrated that the expression of the related target receptor for S100B, RAGE, increased following KA injection and was further attenuated by oral UR and VA. Moreover, mGluR3, an excitatory glutamate receptor, did not participate in this process. Sakatani et al. reported that application of the mGluR3 agonist resulted in a significant increase of extracellular S100B [57]. We suggest that functional activation of mGluR3 is crucial for increasing extracellular S100B instead of protein change.

We previously suggested that oral UR might prevent hippocampal neuronal death from KA treatment by reducing glial cell proliferation. Manley et al. indicated that brain injury-induced epileptic seizures initiate an inflammatory response by potentiating MCP-1 expression and related microglia potentiation during seizure injury within 18 h [58]. Their finding revealed that cytokines play a crucial role in neuroinflammation-related epilepsy [58]. These findings are consistent with our previous findings indicating that auricular electroacupuncture reduces inflammation-related epileptic seizures by altering the TRPA1, pPKC*α*, pPKC*ε*, and pERk1/2 signaling pathways in KA-induced rats [16]. Another study noted an increase in the level of MCP-1 proteins in the hippocampus [59]. Moreover, Lv et al. (2014) indicated that the activation of the NF-*κ*B pathway might contribute to MCP-1 upregulation and microglial activation under epilepsy conditions 24 h after treatment [60]. These results indicated a crucial role of MCP-1 in epileptic seizures. In the current study, the therapeutic period was prolonged up to 6 weeks, mimicking a clinical observation of chronic epilepsy. Our results demonstrated that oral UR treatment for 6 weeks significantly reduced S100B and RAGE overexpression significantly but not that of mGluR3, MCP-1, and CCR-2.

## 5. Conclusion

We established a KA-induced epileptic rat model with KA injections for target research. Our results indicated that KA injection increased the expression of S100B and RAGE in the rat hippocampus. In addition, we reported that oral UR reduced S100B and RAGE overexpression. Similar results were observed after the oral administration of VA, which was used as a positive control. Furthermore, we demonstrated that mGluR3, MCP1, and CCR-2 levels were not altered in all groups, suggesting a negative role in KA-induced epilepsy. Overall, the results implied clear and novel mechanisms for UR treatment on KA-induced epileptic seizures.

## Figures and Tables

**Table 1:** Kainic acid-induced epileptic seizures.

	PBS	KA	UR	VA
Wet-dog shakes	0.0 ± 0.0	309.0 ± 55.1^*∗*^	317.3 ± 47.8^*∗*^	312.8 ± 31.1^*∗*^
Facial myoclonia	0.0 ± 0.0	90.3 ± 12.9^*∗*^	97.8 ± 12.4^*∗*^	93.8 ± 9.4^*∗*^
Paw tremor	0.0 ± 0.0	38.3 ± 4.46^*∗*^	32.8 ± 6.5^*∗*^	38.3 ± 12.0^*∗*^

Mean ± standard deviation. Kainic acid-induced epileptic seizure includes wet-dog shakes, facial myoclonia, and paw tremors. PBS: PBS group; KA: KA group; UR: UR group; VA: VA group; ^*∗*^*P* < 0.05 compared with the PBS group.
